# Regional Variations of Public Perception on Contaminated Industrial Sites in China and Its Influencing Factors

**DOI:** 10.3390/ijerph13040410

**Published:** 2016-04-08

**Authors:** Xiaonuo Li, Wentao Jiao, Rongbo Xiao, Weiping Chen, Yanying Bai

**Affiliations:** 1 State Key Laboratory of Urban and Regional Ecology, Research Center for Eco-Environmental Sciences, Chinese Academy of Sciences, Beijing 100085, China; lixiaonuo1988@163.com (X.L.); wtjiao@rcees.ac.cn (W.J.); wpchen@rcees.ac.cn (W.C.); 2 University of Chinese Academy of Sciences, Beijing 100049, China; 3 Guangdong Provincial Academy of Environmental Science, Guangzhou 510045, China; ecoxiaorb@163.com; 4 Institute of Geographic Sciences and Natural Resources Research, Chinese Academy of Sciences, Beijing 100101, China

**Keywords:** public perception, contaminated site management, soil contamination, environmental management, decision making

## Abstract

Public involvement is critical in sustainable contaminated site management. It is important for China to improve public knowledge and participation, foster dialogue between urban managers and laypeople, and accelerate the remediation and redevelopment processes in contaminated site management. In this study, we collected 1812 questionnaires from nine cities around China through face-to-face interviews and statistically analyzed the perception of residents concerning contaminated sites. The results show that respondents’ concern about soil pollution was lower than for other environmental issues and their knowledge of soil contamination was limited. The risks posed by contaminated industrial sites were well recognized by respondents, but they were unsatisfied with the performance of local agencies regarding information disclosure, publicity and education and public participation. Respondents believed that local governments and polluters should take the primary responsibility for contaminated site remediation. Most of them were unwilling to pay for contaminated site remediation and preferred recreational or public service redevelopment. Moreover, our research indicated that public perception varied among different cities. This variation was mainly determined by implementations of policy instruments and additionally affected by remediation technology, pollutant type, regional policy response and living distance.

## 1. Introduction

Contaminated industrial sites have aroused great concern worldwide due to their significant adverse effects on human health and the environment [1,2,3]. It is necessary to remediate and redevelop these brownfields to mitigate the environmental and public health risk as well as to profitably reuse these sites for urban development [4,5]. In this regard, the Comprehensive Environmental Response Compensation and Liabilities Act (CERCLA), commonly referred to as Superfund [6], is the most successful example in terms of liability, financial responsibility, public participation and other issues involved in contaminated site management. By 2012, more than 1664 sites had been listed on the National Priority List (NPL) and 359 sites had been cleaned up in USA [7].

There is currently an active international debate about how best to ensure that land contamination is managed in a sustainable manner [8]. In this context, sustainable remediation and soft-reuse of brownfields are discussed to balance the relationship between environment, society and economic [9,10,11,12,13,14,15]. Effective stakeholder engagement has been identified as a critical requirement for the optimal application of sustainable remediation strategies and in soft-reuse regeneration more widely [16]. Therein, the available evidence has been examined in a number of studies that local community, who are directly exposed to the contaminated sites, has been considered as the key stakeholder and associated with successful contaminated site management except other core stakeholders [17]. Their considerable attention and social thoughts on contaminated site, as defined by environmental sociology which is typically the sociological study of societal-environmental interactions, can be used to guide solutions to defined environmental problems [18,19]. Reports on the perceptions of residents living on or nearby contaminated sites have burgeoned during the past decades, including residents’ concerns about possible effects of contamination [20,21,22,23,24], willingness to pay for contaminated site remediation [25,26] and factors influencing residents’ perceptions [27,28,29,30]. In general, responses of residents to environmental hazards such as contaminated sites tend to be emotional and residents’ perceptions are affected by complicated factors including subjective variables, trust in the city council, risk information and involvement in risk regulation. Their familiarity with contaminated sites increases as respondents’ distance from the contaminated site decreases [31], and strong preferences of the respondents for land reuse are shown for recreation, cultural and other community facilities while less interest is reported for industrial and business uses [2,32,33,34,35,36]. For sustainable management of contaminated sites, it is necessary to understand public perception and the associated influencing factors, which can help to improve public knowledge and participation, foster dialogues between urban managers and laymen, and accelerate the remediation and redevelopment process.

With the rapid urbanization in past decades, contaminated industrial sites have become a new and serious environmental problem in China. A report officially released by the Environmental Protection Ministry shows that 16 percent of China’s soil and nearly 20 percent of the farmland is polluted [37]. It is estimated that there are more than 200,000 contaminated sites nationwide with cadmium, nickel and arsenic as top pollutants [38,39]. However, this is just the tip of the iceberg. One of the great challenges for site management in China is a lack of public involvement to enhance information transparency, public outreach, liability and supervision mechanisms [40]. Furthermore, studies on residents’ attitudes towards contaminated sites in China are scarce [17].

In this research, we developed a comprehensive questionnaire involving four parts and 34 questions to investigate the perception of residents on contaminated sites. 1812 questionnaires from nine cities around China were collected through face-to-face interviews. Respondents’ perceptions on soil contamination, execution capability of local authorities and preferences for remediation and redevelopment were analyzed. The internal and external factors affecting public perception were further analyzed.

## 2. Materials and Methods

### 2.1. Questionnaire Design

The questionnaire is entitled “Awareness of residents towards soil contamination” and consists of four parts (Table A1). The first part refers to socio-demographic information, including sex, age, education, occupation, household size, income and duration of residence. The second part concerns the basic views of residents towards soil contamination including their attention to, familiarity with and awareness of soil contamination (Q1 to Q9 in Table A1). The third part of the questionnaire is constituted of 14 items with yes-no-neutral choices on residents’ satisfaction with local authorities in terms of information disclosure (Q10 to Q15 in Table A1), publicity and education (Q16 to Q19 in Table A1) and public participation (Q20 to Q23 in Table A1). The fourth part concerns residents’ opinions on contaminated site management policies (Q24 to Q27 in Table A1), impacts caused by, subjective responsibility for and willingness to pay for contaminated site remediation (Q28 to Q30 in Table A1), and respondents’ preferences for alternative reuse possibilities and factors affecting their purchase of dwellings built on remediated land (Q31 to Q34 in Table A1).

### 2.2. Sample Collection

Based on the principles of regional policy response and site representativeness (pollutant type, remediation technology, remediation time and spatial distribution), 18 contaminated sites from nine cities including Beijing (BJ), Chongqing (CQ), Hangzhou (HZ), Shenyang (SY), Wuhan (WH), Lanzhou (LZ), Shanghai (SH), Guangzhou (GZ) and Zhuzhou (ZZ) were chosen to investigate public perception on heavy metal or organic contaminated industrial sites. To make the sample statistically valid, we surveyed 220 residents from each city located within 1.5 km of contaminated sites. A total of 1812 questionnaires (Table 1) were collected through face-to-face interviews between May 2014 and August 2014.

Regarding our study, no content (see the content of Table A1) conflicts with ethical issues. Thus, we did not conduct consultation with an ethical review board. The investigation in this research was conducted through face to face interviews (for the general public) or e-mail communication (for stakeholder professionals). The investigation locations were open to the public, thus no specific permission was obtained. As the investigation locations were randomly selected, GPS positions were not recorded. Before investigation, we clearly introduced our research purposes to all the participants, and let them know that their opinions as a group would be published in scientific journals. All subjects gave their informed consent for inclusion before they participated in the study. We must clarify that the answered questionnaire can be considered as evidence of consent though the consent was not documented, because the participants would not fill out the questionnaire if we didn’t receive their consent. However, the participants only provided socio-demographic information (Part I of Table A1) and did not provide private information such as names and email addresses. The descriptive statistics of respondents’ socio-demographic characteristics are shown in Table 2. 

Of the respondents, the proportion of men was slightly lower than that of women (45.2% *vs*. 54.8%). Their average age was 37% and 25.9% held a bachelor’s degree, while 77.2% had finished high school studies. The majority of the interviewees were working (70%) with the remaining 30% being jobless (9.2%), students (8.1%) or retired (12.7%). The average household size was 3.65 persons and 79.4% of them had low income (less than 800 USD per month). The duration of residence of a larger part of the interviewees (59.6%) was more than 5 years. There were no significant statistical differences in socio-demographic characteristics among the different regions.

### 2.3. Data Analysis

Data were tabulated in Microsoft Office Excel format and statistically analyzed using SPSS 20.0 for Windows. At the initial stage of data analysis, sampling adequacy is tested by KMO and Bartlett’s as 0.806, which means that the questionnaire is with a good construct validity and the data answers are meritorious. Principal Component Analysis (PCA) was used to find internal factors affecting public perception on soil contamination and to compare public perception from various regions. Variance analysis (ANOVA) was implemented to investigate the way in which public perception (dependent variable) was affected by external factors (independent variables) including remediation technology, pollutant type, regional policy response and living distance. The *post hoc* Tamhane’s T2 test and least significant difference (LSD) were applied in multiple comparison tests, respectively, for variables with homogeneity of variance and non-homogeneity of variance.

## 3. Results and Discussion

### 3.1. Perception on Soil Contamination

**Figure 1:**
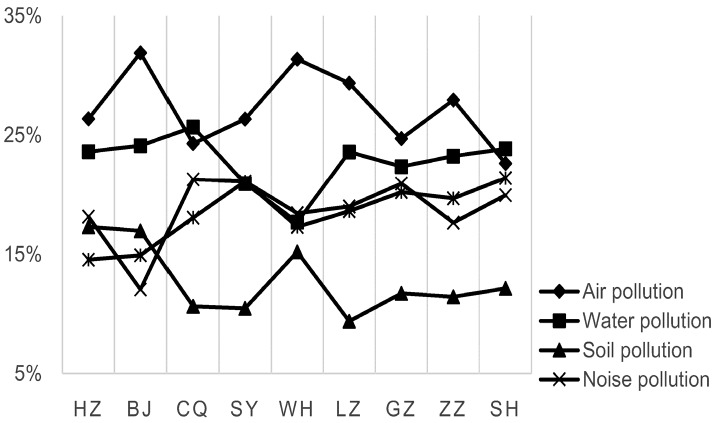
Residents’ attention to environmental problems in different cities.

Figure 1 illustrates the attention paid by respondents to various environmental problems. On average, soil pollution was the primary concern of only 12.81% of the residents, which was much lower than for the other four environmental pollutions—air pollution (27.22%), water pollution (22.80%), noise pollution (18.73%) and waste pollution (18.43%). The results indicate that while the respondents generally recognized soil pollution as a problem, their concern about soil pollution was lower than about other environmental issues due to its hidden properties and relatively lower media exposure. The familiarity with soil contamination was shown to be “low” to “very low” by a majority of the residents, except for Hangzhou and Beijing, where it was moderate to high. These results may be due to two well-known accidents where toxic gases escaped from *in-situ* remediation in a pesticide factory in Hangzhou and a coking plant in Beijing, causing secondary pollution that affected the surrounding residents. Here, secondary pollution means the pollution caused by improper actions during soil remediation process, e.g., toxic gas effusion and residue disposal.

Further investigations into the perceptions of the residents on soil pollution show that 65.1%~88.1% of them had little knowledge about soil contamination (Table 3). However, the majority of the respondents (76%) became aware of the existence of soil pollution adjacent to their houses by media (29.98%~39.82%), website (15.74%~24.43%), chatting with friends (11.05%~20.12%) and communication platforms including wechats and microblogs (7.49%~13.27%). The risks posed by contaminated industrial sites was recognized by more than 97% of the residents and they believed that risks to their health by ingestion and skin contact were more serious than risks to groundwater, crops and the ecological environment. A small number of residents in Hangzhou, Guangzhou and Zhuzhou were given subsidies to relocate away from soil-polluted areas, while exposed people in other cities were not compensated due to flawed policy (34.67%~47.83%), weak management (17.77%~30.62%) and ignored vulnerable groups (19.37%~25.78%). There is also controversy that no compensation due to no evidence on threatened health. Residents in Wuhan (39.94%), Lanzhou (37.67%) and Zhuzhou (42.73%) showed strong willingness to relocate away from contaminated sites, while residents of other cities did not show willingness because of high cost to move and emotional dependence.

### 3.2. Perception on Execution Capability of Local Agencies

**Figure 2:**
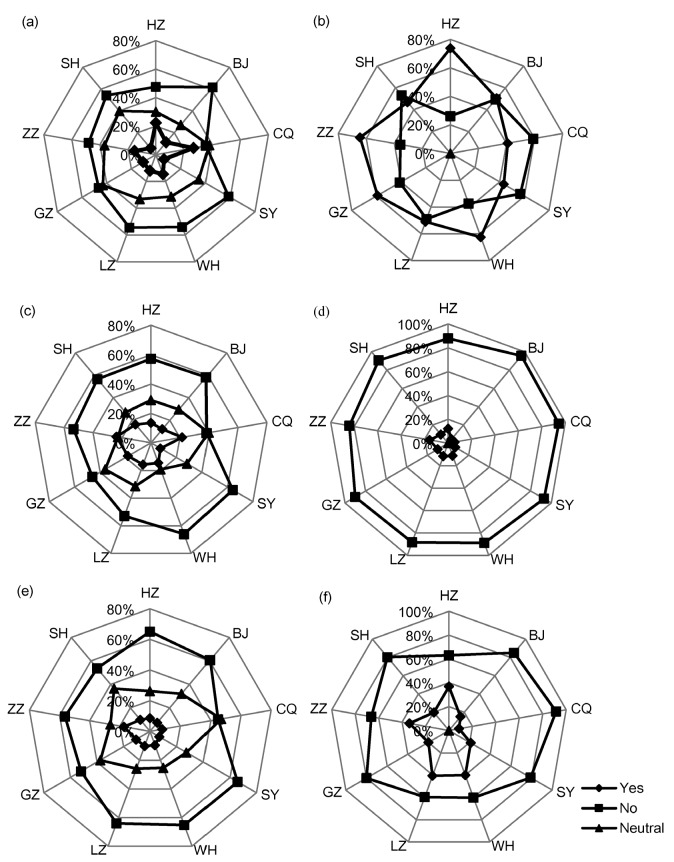
Residents’ response to policy instruments in respect to information disclosure, publicity and education and public participation. (**a**) percent of respondents accessible to information disclosure; (**b**) percent of respondents paying attention to information disclosure; (**c**) percent of respondents accessible to popularization and education activities; (**d**) percent of respondents participating in popularization and education activities; (**e**) percent of respondents accessible to public participation activities; (**f**) percent of respondents involved in public participation activities.

Figure 2 illustrates residents’ perception on execution capability of local agencies including information disclosure, publicity and education and public participation. The majority of residents responded that no actions in any form have ever been conducted disclosing soil contamination information (35.4%–61.7%), promoting knowledge of soil pollution prevention (38.4%–66.2%) or guiding public participation (44.9%–65.7%; see the outermost circle in Figure 2a,c,e). The Connection of these response percentages and the results of residents’ attention to and participation in policy instruments suggested that high disclosure was associated with high concern (Figure 2b). Meanwhile, low availability was significantly associated with low initiative, which led to the phenomenon that 84.2%–95.6% of the residents never took part in activities to promote knowledge on soil pollution prevention (Figure 2d) and 59.6%–91.4% of the residents were not involved in soil remediation and land redevelopment (Figure 2f).

In summary, respondents from all cities were unsatisfied with the performance of local agencies on information disclosure, publicity and education and public participation. 42.2% to 69.2% of the residents in eight cities except Chongqing (40.40% answered “yes”) thought that soil pollution information disclosed was insufficient to explain environmental facts (Table 4, entitled “Enough”), and delayed information disclosure had also threatened the public’s right to know for 40.9%–67.7% of the residents in the eight cities (Table 4, entitled “Timeliness”). Besides, soil pollution information announced by local agencies was suspected of underestimating the soil pollution level (Table 4, entitled “Credibility”). 

There was a disparity between the willingness to be informed and administrative nonfeasance. 70.61% and 65.64% of the residents respectively were unsatisfied with the frequency of knowledge dissemination (Table 4, entitled “Frequency”) and convenience for public participation (Table 4, entitled “Convenience”). Among the nine cities, Chongqing was an exceptional case. Chongqing had established oriented and effective departments to deal with issues engaged in contaminated site management. As a result, it had better performance by the environmental protection department in information disclosure (26.8%), publicity and education (21.7%) and public participation (8.1%), and the highest public satisfaction with policy instruments (average 20.8%) (Table 4). The pathways for information disclosure, publicity and education and public participation preferred by residents did not present significant regional differences. The most popular ways for information disclosure and knowledge popularization were similar to those for knowing about soil pollution. Instead of letters (average 7.2%) and e-mail (average 10.58%), public meetings (average 35.19%), hearings (average 28.61%) and telephone (average 18.42%) were considered the top three ways preferred for public participation due to their characteristics of direct feedback and interactive communication.

### 3.3. Preference for Remediation and Redevelopment

**Figure 3:**
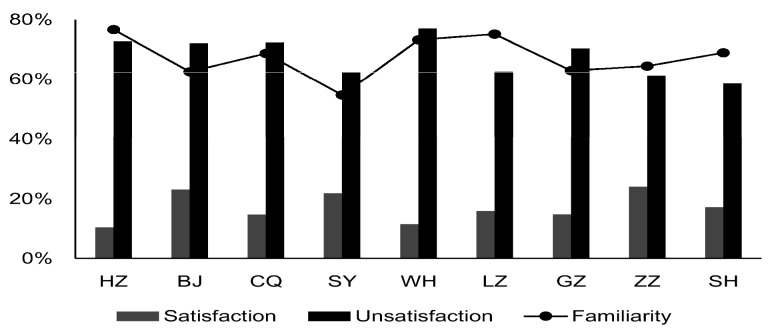
Resident’s response on familiarity and satisfaction with policy.

The investigation on policies for contaminated sites shows that more than half of the respondents had previously heard of such policies. However, there was variation across the cities in the degree of awareness (Figure 3). Respondents in Shenyang (54.7%) were less knowledgeable than the respondents in other cities (62.6%~76.7%). However, respondents showed a lack of confidence in policies. 58.6%~77.01% of the sample were doubtful of policy effectiveness in view of their worries about weak management by local agencies and the infeasibility of policy instruments. The degree of public satisfaction with policies in Zhuzhou (24.1%) and Beijing (23.1%) was much higher than in other cities, which may be attributed to financial support and political tendency toward central government. The pathways preferred by residents to learn about policies were similar to those for knowing about soil pollution.

**Figure 4:**
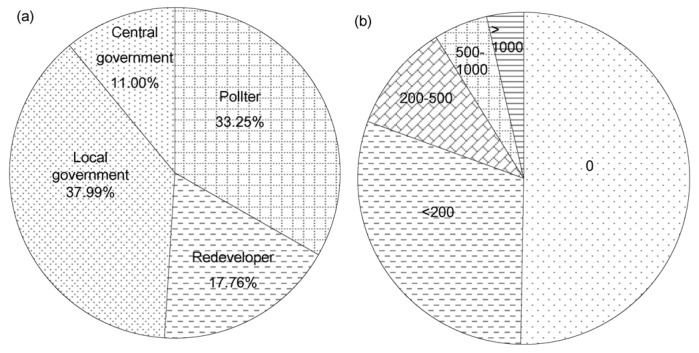
Resident’s response on liability (**a**) and willingness to pay (**b**).

On the subject of liability for contaminated site remediation, 37.99% of residents accused local governments of promoting industries for their economic benefits but failing to supervise their actions, which eventually led to soil contamination (Figure 4a). According to the principle of “polluter pays”, polluting enterprises as the direct dischargers should take the responsibility for contaminated site remediation. Given that the financial conditions of many enterprises in China did not allow them to pay for remediation, only 33.25% of residents thought that the polluter was responsible for soil remediation. A few respondents (11.00%) were of the opinion that the landowner (central government) rather than land user should tackle contaminated site issues, while 17.7% of respondents thought that developers should be responsible for soil remediation.

Public involvement in remediation funding was expected due to the high cost of site remediation. However, more than half of the residents (50.32%) rejected to pay for remediating contaminated sites (Figure 4b), especially those who had suffered or were suffering from secondary pollution (e.g., air pollution, noise pollution and waste pollution) during soil remediation. The others were willing to pay but not for any payment level in the questionnaire. The higher the fees required, the lower the willingness the other respondents had to pay. Only very few interviewees (3.60%) would be willing to pay more than 1000 RMB for contaminated site remediation provided that the living environment was improved by eliminating pollution.

With respect to residents’ acceptance of alternative reuse scenarios, respondents strongly preferred the redeveloped land to provide the public with goods or services (Table 5). 64.28% of the respondents expressed positive judgments for recreational purposes (43.44% as public park, 10.85% as commercial use and 9.99% as play areas), followed by public services (13.60% as transportation, 9.73% as warehouses, 5.11% as nursery and 4.34% as school). Conversely, respondents showed high resistance to residential (36.92%) and agricultural reuse (27.46%), which may pose higher risks to human health. Correspondingly, the majority of the residents hesitated to purchase houses built on remediated land due to subjective factors (25.62%~42.38%) including distrusting the related agencies and worrying about unqualified remediation, economic factors (13.77%~21.77%) including high price and low income, or living factors including comfort (12.44%~18.9%), traffic (9.98%~15.88%), infrastructure (7.73%~12.66%) and location (7.66%~11.69%).

### 3.4. Internal Factors Affecting Public Perception

The questionnaire investigation results illustrate that public perceptions on industrial contaminated sites were generally consistent among the nine cities but had differences for some internal factors. To explain which factors had influence on public perception and the extent in which they affected, PCA was conducted to extract components (with eigenvalue greater than 1) significantly contributing to the variations among cities. Six components involving 22 items were extracted that can totally explain 56.786% of variance (Table 6). Principle component 1 (PC1) included items of Q24, Q22, Q16, Q20, Q25, Q10, Q2, Q13, Q17, Q11, Q14, Q31 and Q21 and can be defined as the factor of policy making and implementation based on the content of these items. Similarly, the other five components can be defined as policy satisfaction factor (PC2, Q26), soil pollution and relocation factor (PC3, Q6, Q9 and Q5), soil pollution hazard factor (PC4, Q4), information disclosure factor (PC5, Q12) and individual willingness factor (PC6, Q33, Q30 and Q17) successively according to the variables they represented. Among the six components, PC1 had the greatest influence on public perception and could explain 21.476% of variance, followed by PC2 (9.634%), PC3 (8.313%), PC4 (6.571%), PC5 (5.627%) and PC6 (5.164%).

In general, the level of public perception in Shanghai, Shenyang, Guangzhou, Lanzhou, Chongqing, Beijing, Wuhan, Zhuzhou and Hangzhou descended according to the PC scores of each city (Table 7). Bothered by serious secondary pollution during the *in-situ* remediation of a pesticide factory, Hangzhou had the lowest score for PC1 and PC2, suggesting that its governmental execution was ineffective and the public dissatisfaction was strong. Compared, as the first city to release specific policy on contaminated site management, Shenyang had the highest PC1 score and the second highest PC2 score. Shenyang was the city providing valuable references on contaminated site policy. It had established explicit departmental responsibilities as well as positive public participation mechanisms through more than ten years’ practical experience. Accordingly, Shanghai ranked 2 in PC1 score and 4 in PC2 score, respectively. The results indicate that the governments of Shenyang and Shanghai effectively responded to contaminated site management and achieved satisfactory performance.

Affected by the secondary pollution accident, residents in Hangzhou showed the highest relocation willingness (highest PC3 score), while residents in Beijing had the lowest relocation willingness because of expensive housing prices. Media exposure of poisonous land in Wuhan (a pesticide factory), Hangzhou (a pesticide factory) and Lanzhou (a petrochemical plant) had drawn public attention to environmental pollution incidents and resulted in higher subjective and emotional concerns about soil pollution hazards, and these cities ranked the top three in PC4 score. In contrast, residents in Shanghai, benefiting from the rich practical experiences without substantial damage by soil contamination, had the lowest PC4 score.

As one of the earliest cities carrying out contaminated site remediation, Chongqing established specialized departments to manage contaminated sites, and had the highest PC5 score, suggesting that Chongqing performed best in information disclosure. Beijing, which was also active in contaminated site information disclosure under pressure of political position and media attention, had the second highest PC5 score. Shanghai and Chongqing ranked the top two in PC6 score, suggesting residents in these cities had higher willingness to purchase houses built on remediated land, to pay for remediation and to participate in publicity activities, which may be attributed to their successful contaminated site management practices. Shenyang had the lowest PC6 score, probably because there was a lack of supporting measures to implement policy instruments.

### 3.5. External Factors Affecting Public Perception

To support the scientific soundness of ANOVA analysis, the delineation of investigated sites including located city, pollutant characteristic, remediation technology, policy response level and neighborhood distance are presented in Table 8.

Based on the results of ANOVA analysis, public perception in the nine cities can be categorized into three groups (Table 9). First, the public perception level of Shenyang (Group I) was found to be significantly higher than that of Hangzhou, Wuhan and Zhuzhou (Group J). The other significant difference was between Shenyang (Group I) and Hangzhou, Beijing, Wuhan and Zhuzhou (Group J). 

Differences between Guangzhou and any other city were insignificant, and the same situation applied to Lanzhou and Chongqing. The results were similar to those of PCA, showing that Shenyang and Shanghai had higher public perception and better contaminated site management. Further analysis indicates that regional policy response, living distance, pollutant type and remediation technology all had significant effects on public perception (*p* < 0.001). The nine cities can be classified in three levels by the number of released policies and implemented instruments. The first level represents more released policies and implemented instruments. Among the cities, Hangzhou, Beijing and Chongqing belong to the first level, Shenyang, Wuhan and Lanzhou belong to the second level, and Guangzhou, Zhuzhou and Shanghai belong to the third level. Inverse to what we expected, public perception in the cities with positive response to national policies (the first level) was lower than those in the cities at the second and third level, suggesting that there was no necessary connection between regional policymaking and policy acquisition.

Residents living in a radius of 0.2 km from contaminated sites had significantly higher perception on soil contamination than those distributed between 1 km and 1.5 km. In comparison to heavy metal contaminated sites, organic contaminated sites had a stronger sensory impact on residents that diffused over a wider range. Accordingly, the perception of the residents living around organic contaminated sites was significantly higher than those around heavy metal contaminated sites. Contaminated sites can be remediated by *ex-situ* and *in-situ* technologies. The *ex-situ* remediation technology involved soil excavation and transportation, which could arouse public focus on soil contamination. As a result, awareness of residents around *ex-situ* remediation sites was higher than those near *in-situ* remediation sites.

## 4. Conclusions

Public involvement is critical and beneficial to facilitate the successful remediation and brownfield site regeneration, while little is known about public perception on contaminated site management in China. A questionnaire survey on residents adjacent to contaminated sites in nine cities around China was carried out. The main findings refined from the representative and science-based raw data are:

1. Respondents are aware of the presence of soil pollution but they pay much lower attention to soil pollution than to air, water, noise and waste pollution. The majority of the respondents recognize the potential health risks posed by industrial contaminated sites. However, they report low willingness to relocate. There was no relationship between health risks and willingness to relocate, suggesting flawed policy and management, and emotional rather than rational public perception.
2. Respondents are unsatisfied with policymaking and policy implementation. The majority of them is doubtful of policy effectiveness and perceives that deficiencies in information disclosure, publicity and education and public participation mechanisms, as well as poor departmental execution and low accountability result in poor perception on management policies.
3. Preference for liability is interconnected with payment preference. Local governments and polluters are thought to be prior to take responsibility for remediating contaminated sites and the majority of residents reject payment. While, some of them are willing to pay a very small amount of money provided the improvement of living environment. Subjective consideration plays an important role in preference for buying houses and land reuse types. The regeneration of land to provide the public with goods or services (e.g., public parks, commercial use and play areas) is preferred, while residential use and agricultural use are strongly opposed.
4. Approaches including media (newspaper and TV), websites, communication platforms (webchats and microblogs) and interactive modes (public meetings, hearings and telephone) don’t show significant regional differences and are preferred in information disclosure, publicity and education and public participation.
5. Public perception in the nine surveyed cities varies in respect to management policy, relocation willingness, risk awareness, information disclosure and individual willingness, and follows a general descending order of Shanghai, Shenyang, Guangzhou, Lanzhou, Chongqing, Beijing, Wuhan, Zhuzhou and Hangzhou, due to the regional heterogeneity in remediation methods, media attention and practice experiences.
6. Public perception was significantly different among cities and was affected by remediation technology, pollutant type, regional policy response and living distance. The perception of residents under the characteristics of *in-situ* remediation, heavy metal pollutants, and living distance between 1 km and 1.5 km were lower than those under the circumstances of the *ex-situ* remediation, organic pollutants, and living within 0.2 km.

The outcomes obtained in this research should be helpful to address issues related to contaminated site management and provide science-based support for future policy making in China. The findings in our study suggest that for sustainable contaminated site management, local governments should give priority to increasing public awareness as well as effective implementation of policy instruments to build trust in local authorities, such as disclosing reliable soil pollution information in a timely manner, popularizing knowledge on preventing soil pollution and establishing available approaches for public accessibility. Furthermore, contaminated site management is a complicated project that involves multiple stakeholders. To avoid potential controversy and disagreement among stakeholders, decision making on policy formulation and implementation, especially liability, payment and land reuse options, should also take public considerations into account, in particular, more attention to those living within 0.2 km of sites polluted by organic pollutants with *ex-situ* remediation technologies.

## Figures and Tables

**Table 1:** Questionnaire distribution and collection.

City	Sites	Distribution	Collection	Availability
Beijing	A coking plant and Songjiazhuang site	220	206	93.6%
Chongqing	A steel factory and a chemical factory	220	198	90%
Hangzhou	A paint factory and a pesticide factory	220	206	93.6%
Shenyang	A coking plant and a storage battery factory	220	200	90.9%
Wuhan	A pesticide factory and a dyestuff factory	220	198	90%
Lanzhou	A petrochemical company	220	202	91.8%
Shanghai	Disneyland site and a chemical plant	220	201	91.4%
Guangzhou	A nitrogen fertilizer factory	220	203	92.3%
Zhuzhou	Qingshuitang site and Liyu site	220	198	90%

**Table 2:** Sample characteristics.

Variables	Options	Percent (%)	Variables	Options	Percent (%)
Sex	Male	45.2	Occupation	Jobless	9.2
	Female	54.8		Student	8.1
Age	<23	13.9		Freelancer	16.2
	23–35	38.5		Worker	37.8
	35–50	28.6		Employer	4.7
	>50	19.0		Professionals	11.3
Education	Below junior high school	22.8	Monthly income in RMB (Yuan)	Retiree	12.7
	High school	31.6		<3000	44.7
	Junior college	19.6		3000–5000	34.7
	Bachelor	21.5		5000–8000	13.1
	Master or above	4.4		>8000	7.6
Household size	1	1.1	Residence time (Year)	<1	10.6
	2	6.3		1–3	13.7
	3	49.2		3–5	16.1
	4	19.5		5–10	15.8
	5	17.5		>10	43.8
	>5	6.4			

**Table 3:** Residents’ perception on soil contamination.

Question	Preferred Option	City
I know about soil contamination very well.	Neutral (34.8%–52.4%)	HZ, BJ, CQ, WH, GZ, ZZ, SH
	Disagree somewhat (35.9%–43.3%)	SY, LZ
I am aware of the contaminated soil on my parcel.	Yes (76.70%–92.70%)	HZ, BJ, CQ, SY, WH, LZ, GZ, ZZ, SH
I know the reason for soil contamination.	Yes (61.16%–83.82%)	HZ, BJ, CQ, SY, WH, LZ, GZ, ZZ, SH
I think that soil contamination has posed risks for the environment and health.	Yes (97.79%–99.74%)	HZ, BJ, CQ, SY, WH, LZ, GZ, ZZ, SH
I have been compensated for soil contamination; if not, the reason.	No, policy (34.65%–47.83%)	HZ, BJ, CQ, SY, WH, LZ, GZ, ZZ, SH
I will relocate to unpolluted region.	Neutral (32.01%–40.07%)	HZ, SY, WH, LZ, GZ, ZZ, SH
	No (37.42%–42.37%)	BJ, CQ

**Table 4:** Residents’ perception on execution capability of local agencies.

Perception		HZ	BJ	CQ	SY	WH	LZ	GZ	ZZ	SH
Enough	Yes	11.20%	17.50%	40.40%	2.50%	7.10%	6.10%	6.50%	7.40%	2.50%
	No	63.60%	42.20%	19.70%	63.20%	69.20%	64.60%	64.50%	62.40%	67.50%
	Neutral	25.20%	40.30%	39.90%	34.30%	23.70%	29.30%	29.00%	30.20%	30.00%
Timeliness	Yes	7.80%	3.40%	23.20%	3.50%	6.60%	5.10%	7.00%	6.40%	2.00%
	No	67.50%	66.50%	40.90%	66.20%	67.70%	66.70%	64.50%	64.90%	64.00%
	Neutral	24.80%	30.10%	35.90%	30.30%	25.80%	28.30%	28.50%	28.70%	34.00%
Credibility	Yes	21.80%	7.30%	22.20%	28.90%	23.20%	32.30%	20.50%	27.70%	32.50%
	No	36.40%	68.90%	43.90%	32.80%	34.30%	15.20%	34.00%	34.20%	25.10%
	Neutral	41.70%	23.80%	33.80%	38.30%	42.40%	52.50%	45.50%	38.10%	42.40%
Frequency	Yes	3.90%	2.90%	12.60%	3.00%	3.50%	7.10%	8.00%	7.40%	6.90%
	No	74.80%	79.60%	53.00%	76.60%	74.20%	65.20%	63.00%	72.30%	76.80%
	Neutral	21.40%	17.50%	34.30%	20.40%	22.20%	27.80%	29.00%	20.30%	16.30%
Convenience	Yes	5.30%	3.40%	5.60%	3.00%	5.60%	4.50%	6.00%	9.40%	3.90%
	No	67.50%	75.20%	57.60%	68.70%	70.20%	66.70%	54.00%	62.40%	68.50%
	Neutral	27.20%	21.40%	36.90%	28.40%	24.20%	28.80%	40.00%	28.20%	27.60%

**Table 5:** Public acceptance of alternative reuse scenarios.

Reuse Type	Strongly Agree (%)	Acceptable (%)	Reluctance (%)
Agriculture	1.87	1.99	27.46
Commercial use	10.85	19.49	3.51
Warehouse	9.73	14.02	10.51
Transportation	13.60	15.26	5.15
Play areas	9.99	15.35	5.16
Residential use	1.07	2.06	36.92
Nursery	5.11	13.74	6.09
School	4.34	14.69	4.61
Public park	43.44	3.41	0.60

**Table 6:** Principal components loading.

Definition	Variable	Component					
		PC1	PC2	PC3	PC4	PC5	PC6
Familiarity with policies	X17	0.640	−0.492	0.318	−0.142	−0.130	−0.090
Satisfaction with public participation	X16	0.572	0.308	0.011	−0.273	0.283	−0.023
Popularization or not	X11	0.565	0.220	0.024	−0.227	0.348	0.006
Soliciting opinions or not	X14	0.552	0.274	0.004	−0.286	0.316	−0.048
Willingness to learn about policies	X19	0.541	−0.417	0.375	−0.164	−0.223	−0.208
Information disclosure or not	X6	0.540	0.379	−0.002	0.153	−0.262	−0.077
Familiarity with soil pollution	X1	0.534	−0.207	0.079	0.502	0.230	0.159
Timeliness of information disclosure	X9	0.522	0.501	−0.019	0.142	−0.443	−0.048
Satisfaction with popularization	X13	0.520	0.334	−0.018	−0.241	0.271	−0.092
Attention on information disclosure	X7	0.511	−0.036	−0.082	0.348	0.053	0.082
Credibility of information disclosure	X10	0.441	0.251	0.083	−0.151	−0.055	0.067
Satisfaction with redevelopment	X21	0.420	−0.043	−0.089	−0.256	−0.014	0.148
Public participation	X15	−0.309	0.262	0.019	−0.080	−0.224	−0.131
Satisfaction with policies	X18	−0.524	0.582	−0.382	0.185	0.200	0.190
Soil pollution reason	X4	−0.201	0.257	0.737	0.250	0.164	0.018
Willingness to relocate	X5	−0.227	0.318	0.725	0.092	0.075	0.084
Extent of soil contamination	X3	0.568	−0.232	−0.574	0.168	0.008	−0.040
Soil pollution hazards	X2	0.540	−0.147	0.075	0.550	0.215	0.183
Extent of information disclosure	X8	0.479	0.457	−0.023	0.197	−0.486	0.002
Willingness to purchase	X22	0.057	−0.032	0.048	−0.346	−0.155	0.618
Willingness to pay	X20	−0.065	0.077	−0.062	0.152	0.204	−0.609
Extent of participating popularization	X12	0.082	−0.068	0.007	0.062	0.037	0.398

**Table 7:** Principle component scores of different cities.

Region	PC1	Rank	PC2	Rank	PC3	Rank	PC4	Rank	PC5	Rank	PC6	Rank	F	Rank
HZ	−0.365	9	−0.229	9	0.237	1	0.108	2	−0.080	8	0.126	3	−0.126	9
BJ	−0.035	5	−0.005	5	−0.299	9	−0.027	6	0.078	2	0.045	4	−0.049	6
CQ	−0.056	6	−0.097	8	−0.194	8	−0.080	7	0.246	1	0.130	2	−0.039	5
SY	0.448	1	0.135	2	−0.084	7	−0.131	8	−0.073	7	−0.287	9	0.131	2
WH	−0.330	8	−0.051	6	0.140	3	0.168	1	−0.049	6	0.034	5	−0.095	7
LZ	0.026	4	0.161	1	0.201	2	0.093	3	−0.018	4	−0.040	6	0.072	4
GZ	0.166	3	0.105	3	0.036	5	0.019	4	−0.040	5	−0.076	7	0.077	3
ZZ	−0.185	7	−0.092	7	0.042	4	−0.011	5	−0.089	9	−0.216	8	−0.109	8
SH	0.332	2	0.080	4	−0.072	6	−0.135	9	0.028	3	0.279	1	0.141	1

**Table 8:** Basic information on investigated sites.

City	Site	Pollutant	Remediation Technology	Policy Level	Distance
Hangzhou	A paint factory	Heavy metals	Ex-site	1st level	1000–1500 m
	A pesticide factory	Organic pollutants	In-site	1st level	<100 m
Beijing	A coking plant	Organic pollutants	In-site	1st level	<100 m
	Songjiazhuang site	Organic pollutants	Ex-site	1st level	200–400 m
Chongqing	A chemical factory	Organic pollutants	Ex-site	1st level	200–400 m
	A steel factory	Heavy metals	Ex-site	1st level	700–1000 m
Shenyang	A Coking plant	Organic pollutants	Ex-site	2nd level	400–500 m
	A storage battery factory	Heavy metals	Ex-site	2nd level	500–700 m
Wuhan	A pesticide factory	Organic pollutants	In-site	2nd level	400–500 m
	A dyestuff factory	Heavy metals	In-site	2nd level	200–400 m
Lanzhou	A petrochemical company	Organic pollutants	Ex-site	2nd level	200–400 m
Guangzhou	A nitrogen fertilizer factory	Organic pollutants	Ex-site	3rd level	<100 m
Zhuzhou	Liyu site	Heavy metals	Ex-site	3rd level	200–400 m
	Qingshuitang site	Heavy metals	In-site	3rd level	1000–1500 m
Shanghai	Disneyland site	Heavy metals	Ex-site	3rd level	1000–1500 m
	A chemical plant	Organic pollutants	Ex-site	3rd level	<100 m

**Table 9:** Results of ANOVA on public perception.

Method	Variable	(I)	(J)	Mean Difference (I–J)	Significance
Tamhane’s T2	City	Shenyang	Hangzhou	0.258 *	0.003
			Wuhan	0.227 *	0.012
			Zhuzhou	0.240 *	0.003
		Shanghai	Hangzhou	0.267 *	0.001
			Beijing	0.190 *	0.030
			Wuhan	0.237 *	0.005
			Zhuzhou	0.250 *	0.001
	Policy	1st level	2nd level	−0.108 *	0.013
			3rd level	−0.108 *	0.011
	Distance	1000–1500 m	<200 m	−0.206 *	0.000
LSD	Pollutant	Heavy metal	Organic pollutant	−0.146 *	0.000
	Technology	*In-situ*	*Ex-situ*	−0.255 *	0.000

* The mean difference is significant at 0.05 level.

## Appendix A

**Table A1:** Awareness of residents towards soil contamination.

**Part I. Socio-Demographic Information**		
**Items**		**Options**
Sex		□Male □Female
Age (Year)		□<23 □23–35 □35–50 □>50
Education		□Below junior high school □High school □Junior college □Bachelor □Others
Occupation		□Jobless □Student □Freelancer □Worker □Employer □Professionals □Retiree
Household size		□1 □2 □3 □4 □5 □>5
Monthly income in RMB (Yuan)		□<3000 □3000–5000 □5000–8000 □>8000
Duration of residence (Year)		□<1 □1–3 □3–5 □5–10 □>10
**Part II. Questions on Soil Contamination.**		
**Items**		**Options**
Q1	The pollution problem you concern most.	□Air □Water □Soil □Noise □Wastes
Q2	I know about soil contamination very well.	□Agree strongly □Agree somewhat □Neutral □Disagree somewhat □Disagree strongly
Q3	The ways that you are acquainted with soil contamination.	□Websites □Media (newspaper and TV) □Communication platform (wechat and microblog) □Popularization of science □Materials issuing □Chat □Bulletin □Conference □Work
Q4	I know about hazards of soil contamination very well.	□Agree strongly □Agree somewhat □Neutral □Disagree somewhat □Disagree strongly
Q5	I am aware of the contaminated soil on my parcel.	□Yes □No □Neutral
Q6	I know the reason of soil contamination.	□Yes □No
Q7	I think that soil contamination has posed risks for environment and health.	□Yes □No
Q8	I have been compensated for soil contamination, if not, the reason.	□Yes □No, policy □No, executor □No, victim □No, ignorance
Q9	I will relocate in unpolluted region.	□Yes □No □Neutral
**Part III. Questions on Execution Capability of Local Agencies**		
**Items**		**Options**
Q10	Soil pollution information were available to me.	□Yes □No □Neutral
Q11	I have been concerned about soil pollution information.	□Yes □No □Neutral
Q12	Information disclosed is adequate.	□Yes □No □Neutral
Q13	Information disclosed is timely.	□Yes □No □Neutral
Q14	Information disclosed is reliable.	□Yes □No □Neutral
Q15	The ways that are most effective to disclose information.	□Website □Materials issuing □Press conference □Media (newspaper and TV) □Bulletin □Information retrieval location □Communication platform (wechat and microblog)
Q16	Environmental protection departments conducted educational activities on soil pollution hazards and prevention.	□ Yes □No □Neutral
Q17	I have attended educational activities on soil pollution issues.	□Yes □No □Neutral
Q18	The frequency of knowledge popularity is enough.	□Yes □No □Neutral
Q19	The ways that are most effective for dissemination.	□Website □Bulletin □Conference □Popularization of science □Media (newspaper and TV) □Communication platform (wechat and microblog) □Materials issuing
Q20	Environmental protection departments collected public suggestions on contaminated site management.	□Yes □No □Neutral
Q21	I proposed some opinions on contaminated site management.	□Yes □No □Neutral
Q22	The ways to participate in site management are convenient.	□Yes □No □Neutral
Q23	The ways that are most effective for public participation.	□Email □Telephone □Letter □Seminar □Hearing
**Part IV. Questions on Policy, Remediation and Redevelopment**		
**Items**		**Options**
Q24	I am acquainted with contaminated site management policies.	□Agree strongly □Agree somewhat □Neutral □Disagree somewhat □Disagree strongly
Q25	I want to know more about contaminated site management policies.	□Yes □No □Neutral
Q26	I am satisfied with contaminated site management policies, e.g., segments on information disclosure and sustainable remediation.	□Yes □No □Neutral
Q27	The most effective approaches to know about contaminated site management policies:	□Website □Bulletin □Conference □Popularization of science □Media (newspaper and TV) □Communication platform (wechat and microblog) □Materials issuing
Q28	My normal life is disturbed by soil remediation project in terms of:	□No □Noise pollution □Air pollution □Wastes pollution □Traffic jam □Aesthetic impact
Q29	I think soil remediation project should be funded by:	□Polluter □Developer □Local government □Central government
Q30	I’m willing to pay for soil remediation with certain amount of money.	□0 □<200 RMB □200–500 RMB □500–1000 RMB □>1000 RMB
Q31	I am satisfied with the current type of land regeneration.	□Yes □No □Neutral
Q32	I prefer land regeneration to these possibilities:	□Agriculture □Commercial use □Warehouse □Transportation □Play areas □Residential use □Nursery □Scholl □Public park
Q33	I’m willing to buy a house on brownfield site after remediated.	□Yes □No □Neutral
Q34	My considerations on houses on brownfield site after remediated:	□Infrastructure □Price □Location □Traffic □Convenience □Distrustful remediation outcome

## References

[1] Bergius K., Öberg T. (2007). Initial screening of contaminated land: A comparison of U.S. and Swedish methods. J. Environ. Manag..

[2] Greenberg M., Lewis M.J. (2000). Brownfields redevelopment, preferences and public involvement: A case study of an ethnically mixed neighborhood. Urban Stud..

[3] Van Straalen N.M. (2002). Assessment of soil contamination—A functional perspective. Biodegradation.

[4] Bromberg L.M., Spiesman T. (2006). Turning an economic liability into an asset: The anatomy of a redevelopment project. N. J. Law J..

[5] Xie J., Li F.S. Overview of the Current Situation on Brownfield Remediation and Redevelopment in China. http://documents.worldbank.org/curated/en/2010/09/13132932/overview-current-situation-brownfield-remediation-redevelopment-china.

[6] United States Environmental Protection Agency (USEPA) Comprehensive Environmental Response, Compensation, and Liability Act (CERCLA), 1980. http://www.epw.senate.gov/cercla.pdf.

[7] United States Environmental Protection Agency (USEPA) (2012). EPA Adds Three Hazardous Waste Sites to Superfund’s National Priorities List. http://yosemite.epa.gov/opa/admpress.nsf/0/c9b5998d8d45e168852579f8005c9453.

[8] Bardos P. (2014). Progress in Sustainable Remediation. Remediat. J..

[9] Bardos P., Bone B., Boyle R., Ellis D., Evans F., Harries N.D., Smith J.W.N. (2011). Applying sustainable development principles to contaminated land management using the surf-uk framework. Remediat. J..

[10] Bardos P., Bone B.D., Boyle R., Evans F., Harries N.D., Howard T., Smith J.W.N. (2016). The rationale for simple approaches for sustainability assessment and management in contaminated land practice. Sci. Total Environ..

[11] Bardos P., Jones S., Stephenson I., Menger P., Beumer V., Neonato F., Maring L., Ferber U., Track T., Wendler K. (2015). Optimising value from the soft re-use of brownfield sites. Sci. Total Environ..

[12] Bardos P., Stephenson I., Menger P., Beumer V. (2015). Maximising the value-proposition for soft re-use of brownfields. AquaConsSoil 2015, Session 2.3—Redevelopment of brownfields 1. http://www.zerobrownfields.eu/HombreMainGallery/Docs/Bardos%20Session%202.3%20ACS%202015%20v2%20Hombre%20web%20site.pdf.

[13] Beumer V., Bardos P., Menger P. HOMBRE D 5.2: Decision Support System on Soft Reuses. HOMBRE Deliverable D 5-2. Deltares, 2014. http://www.zerobrownfields.eu/HombreTraining.

[14] Cundy A., Bardos P., Puschenreiter M., Witters N., Mench M., Bert V., Friesl-Hanl W., Müller I., Weyens N., Vangronsveld J. (2015). Developing effective decision support for the application of “gentle” remediation options: The GREENLAND project. Remediat. J..

[15] Contaminated Land: Applications in Real Environments (CL: AIRE) The SuRF-UK Indicator Set for Sustainable Remediation Assessment. http://www.claire.co.uk/index.php?option=com_content&view=article&id=748:annex-1-surf-uk-indicator-set-for-sustainable-remediation&catid=966&Itemid=78.

[16] Cundy A.B., Bardos R.P., Church A., Puschenreiter M., Friesl-Hanl W., Müller I., Neu S., Mench M., Witters N., Vangronsveld J. (2013). Developing principles of sustainability and stakeholder engagement for "gentle" remediation approaches: The European context. J. Environ. Manag..

[17] Li Y.Y., Tan S.K. (2012). Cognition of Residents and Influencing Factors around Wuhan. China Real Estate.

[18] Scarce R. (2009). Social Theories of the Environment. http://www.skidmore.edu/~rscarce/Soc-Th-Env/Env%20Theory%20PDFs/2009EnvTheorysyllabus.pdf.

[19] Wikipedia. https://en.wikipedia.org/wiki/Environmental_sociology.

[20] Eiser J.R., Stafford T., Henneberry J., Catney P. (2007). Risk perception and trust in the context of urban brownfields. Environ. Hazards.

[21] Eiser J.R., Stafford T., Henneberry J., Catney P. (2009). “Trust me, I’m a Scientist (Not a Developer)”: Perceived expertise and motives as predictors of trust in assessment of risk from contaminated land. Risk Anal..

[22] Vandermoere F. (2006). The process of soil excavation in a community: Site-specific determinants of stress perception. Environ. Behav..

[23] Scholz R.W., Siegrist M. (2010). Low risks, high public concern? The cases of Persistent Organic Pollutants (POPs), heavy metals, and nanotech particles. Hum. Ecol. Risk Assess..

[24] Alberini A., Tonin S., Turvani M. (2007). Willingness to pay for contaminated site cleanup policies: Evidence from a conjoint choice study in Italy. Rev. D’économie Polit..

[25] Alberini A., Tonin S., Turvani M., Chiabai A. (2007). Paying for permanence: Public preferences for contaminated site cleanup. J. Risk Uncertain..

[26] Dunn J.R., Taylor S.M., Elliott S.J., Walter S.D. (1994). Psychosocial effects of PCB contamination and remediation: The case of Smithville, Ontario. Soc. Sci. Med..

[27] Matthies E., Höger R., Guski R. (2000). Living on polluted soil: Determinants of stress symptoms. Environ. Behav..

[28] Vandermoere F. (2008). Hazard perception, risk perception, and the need for decontamination by residents exposed to soil pollution: The role of sustainability and the limits of expert knowledge. Risk Anal..

[29] Vandermoere F. (2008). Psychosocial health of residents exposed to soil pollution in a Flemish neighborhood. Soc. Sci. Med..

[30] Grasmück D., Scholz R.W. (2005). Risk perception of heavy metal soil contamination by high exposed and low-exposed inhabitants: The role of knowledge and emotional concerns. Risk Anal..

[31] Tonin S., Turvani M., Alberini A. (2011). Knowledge about, importance of, and attitudes towards industrial brownfield re-use. Sci. Reg..

[32] Feldman D.L., Hanahan R.A. (1996). Public perceptions of a radioactively contaminated site: Concerns, remediation preferences, and desired involvement. Environ. Health Perspect..

[33] Turvani M., Chiabai A., Alberini A., Tonin S. (2006). Public Support for Policies Addressing Contaminated Sites: Evidence from a Survey of the Italian Public. http://www.researchgate.net/profile/Aline_Chiabai/publication/23732189_Public_Support_for_Policies_Addressing_Contaminated_Sites_Evidence_From_a_Survey_of_the_Italian_Public/links/0fcfd50752bb23cb6c000000.pdf.

[34] Turvani M., Paccagnan V., Tonin S. (2006). Population Preferences towards Risk and Alternative Reuse Policies for Derelict and Contaminated Sites: Results from a Survey of the Italian Public. http://www.kent.ac.uk/scarr/events/beijingpapers/Turvanippr.pdf.

[35] Alberini A., Aline C., Turvani M., Tonin S. (2007). Public Policies for Contaminated Site Cleanup: The Opinions of the Italian. http://ssrn.com/abstract=962378.

[36] Weber O., Scholz R.W., Bühlmann R., Grasmück D. (2001). Risk perception of heavy metal soil contamination and attitudes to decontamination strategies. Risk Anal..

[37] China Daily (2015). A Bumpy Road to Clean up China’s Contaminated Land. http://www.chinadaily.com.cn/china/2015-06/25/content_21099483.htm.

[38] BBC News (2014). Report: One Fifth of China’s Soil Contaminated. http://www.bbc.co.uk/news/world-asia-china-27076645.

[39] Everbright Securities (ES) (2013). Soil Remediation: 700 billion “Feast” under Policy Approaching. http://pg.jrj.com.cn/acc/Res/CN_RES/INDUS/2013/9/3/eb13500d-027d-48e6-854d-667e5f262baf.pdf.

[40] Li X.N., Jiao W.T., Xiao R.B., Chen W.P., Chang A.C. (2015). Soil pollution and site remediation policies in China: A review. Environ. Rev..

